# Characterizing the highest tropical cyclone frequency in the Western North Pacific since 1984

**DOI:** 10.1038/s41598-021-93824-2

**Published:** 2021-07-12

**Authors:** Joseph Basconcillo, Eun-Jeong Cha, Il-Ju Moon

**Affiliations:** 1 Typhoon Research Center, Jeju National University, Jeju, South Korea; 2 Department of Science and Technology, Philippine Atmospheric, Geophysical, and Astronomical Services Administration, Quezon City, Philippines; 3 National Institute of Meteorological Sciences, Jeju, South Korea

**Keywords:** Atmospheric science, Atmospheric dynamics, Climate sciences, Natural hazards

## Abstract

The 2018 boreal summer in the Western North Pacific (WNP) is highlighted by 17 tropical cyclones (TC)—the highest record during the reported reliable years of TC observations. We contribute to the existing knowledge pool on this extreme TC frequency record by showing that the simultaneous highest recorded intensity of the WNP summer monsoon prompted the eastward extension of the monsoon trough and enhancement of tropical convective activities, which are both favorable for TC development. Such changes in the WNP summer monsoon environment led to the extreme TC frequency record during the 2018 boreal summer. Meanwhile, the highest record in TC frequency and the intensity of the WNP summer monsoon are both attributed with the combined increase in the anomalous westerlies originating from the cold tropical Indian Ocean sea surface temperature (SST) anomalies drawn towards the convective heat source that is associated with the warm central Pacific SST anomalies. Our results provide additional insights in characterizing above normal tropical cyclone and summer monsoon activities in the WNP in understanding seasonal predictable horizons in the WNP, and in support of disaster risk and impact reduction.

## Introduction

**Figure 1:**
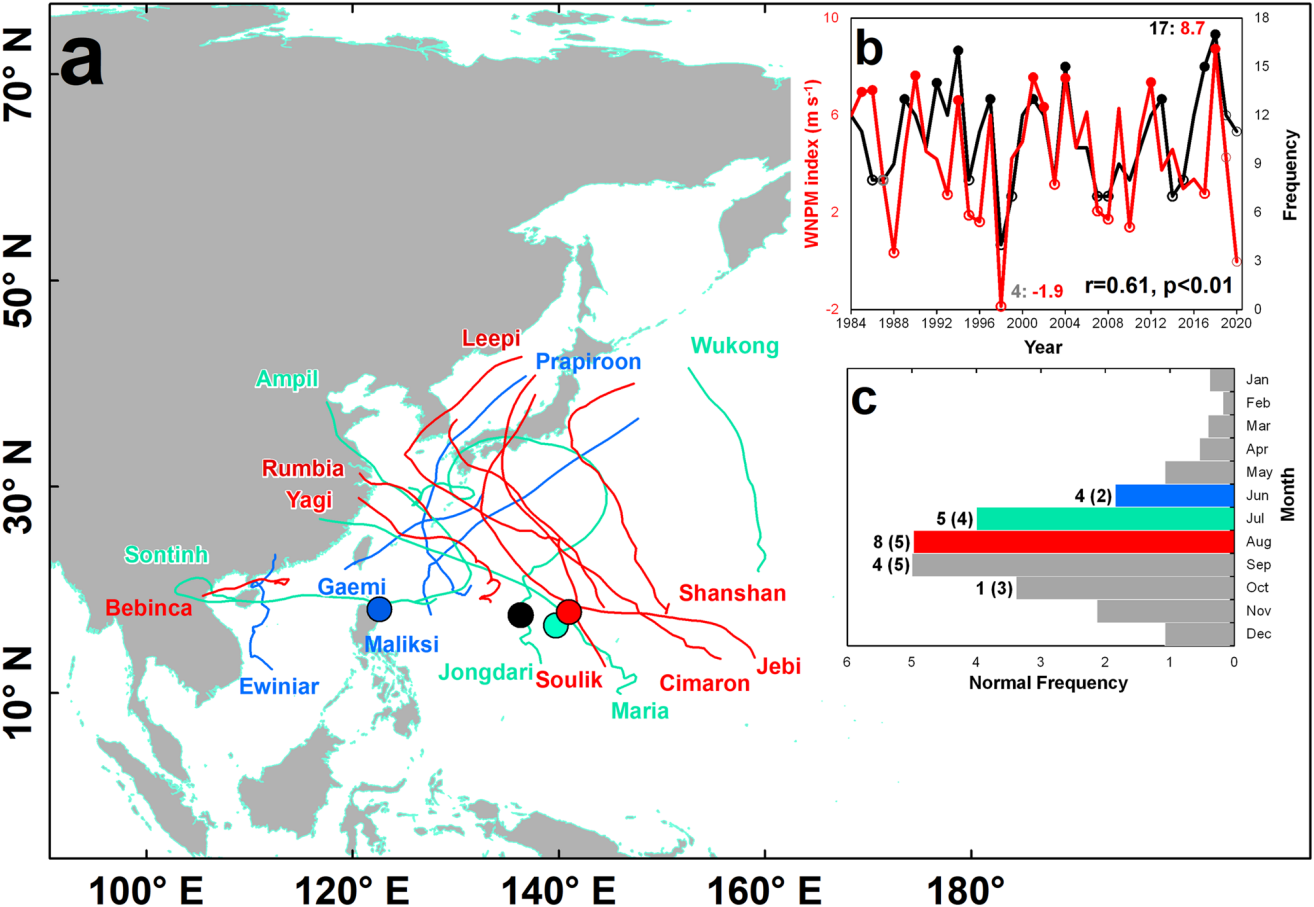
Tropical cyclone (TC) climatology in June–July–August (JJA) in the Western North Pacific (WNP). (**a**) TC tracks during the 2018 JJA. (**b**) Timeseries of the WNP TC frequency during the boreal summer (black) and the WNP summer monsoon index (red). (**c**) Monthly TC frequency in the WNP. In (**a**) and (**c**) the blue, cyan, and red colors represent the monthly TC tracks and TC frequency during June, July, and August, respectively. In (**b**) the inset numbers represent the indicated index values in JJA 2018 (1998)—its index’s highest (lowest) record since 1984. The (filled) dots represent values that are (greater than or equal to the 3rd) less than or equal to the 1st interquartile range values of the index’s climatology, which are the years used in the composite analysis. In (**c**) the inset values outside and inside the parentheses indicate the TC frequency during the 2018 boreal summer and the normal TC frequency. The map is plotted using ArcGIS 10.1 (https://www.esri.com).

There were 17 tropical cyclones (TC) that formed in the Western North Pacific (WNP) basin during the 2018 boreal summer (i.e. June–July–August, JJA)—the highest recorded TC frequency since 1984 (Fig. 1a,b). Moreover, all months in JJA 2018 also recorded TC frequencies that are 100% (June, 4 counts), 25% (July, 5 counts), and 60% (August, 8 counts) above their normal values, respectively (Fig. 1c).

Questions were expectedly raised on what caused such unprecedented increased TC frequency in the WNP hence it is not surprising that a number of reports have already contributed to the existing knowledge pool^1–4^. Of the said reports, the first two studies investigated the TC frequency in the entire North Pacific (i.e. Western and Eastern North Pacific basins), but both reports differ in their choice of seasonal scaling. The first paper used July–November^1^ while the second report selected June–September^2^. Meanwhile, the other two papers focused on the WNP TC frequency but they also differ in their choice of seasonal scaling. The third paper selected June–November^3^ while the most recent report divided the WNP TC frequency into two seasons: JJA and September–October^4^. However, these choices of regional scaling and seasonal clustering do not totally account for the variability that is inherent to each basin and season. For example, we show that the WNP TC frequency in JJA has no significant correlation with the TC frequency in the Eastern North Pacific in the same season (r = 0.22) (Supplementary Fig. S1a,b). Provided that both basins have indeed above normal TC frequency in JJA 2018, the Eastern North Pacific, however, did not register its highest TC frequency record. In addition, we highlight that the WNP TC frequency in JJA has practically no correlation with the WNP TC frequency in September (r = 0.07) and September–November (r = 0.06), respectively (Supplementary Figs. S1c,d). Moreover, the WNP TC frequency in September and September–November 2018 are both below normal and are not their respective season’s extreme records as well. Such differences are largely due to the inherent seasonal changes in the WNP environment, which include the decay of the WNP summer monsoon, onset of the WNP boreal winter monsoon, and seasonal reversal related to the phase changes of the El Niño–Southern Oscillation (ENSO)^5^. For these reasons, we select the WNP and JJA as our regional domain and seasonal scale of analysis, respectively. While our choice of scaling is similar to the latest report^4^, we demonstrate in the succeeding discussions that our conclusion deviates from their reported primary cause of the highest tropical cyclone frequency record in JJA 2018. In particular, we iterate that our aim is to contribute (rather than to discount the merit of the said previous reports) to the existing knowledge pool in understanding the large-scale drivers behind above normal TC frequency and activity in the WNP.

## Results

### Large-scale environment anomalies in JJA 2018

**Figure 2:**
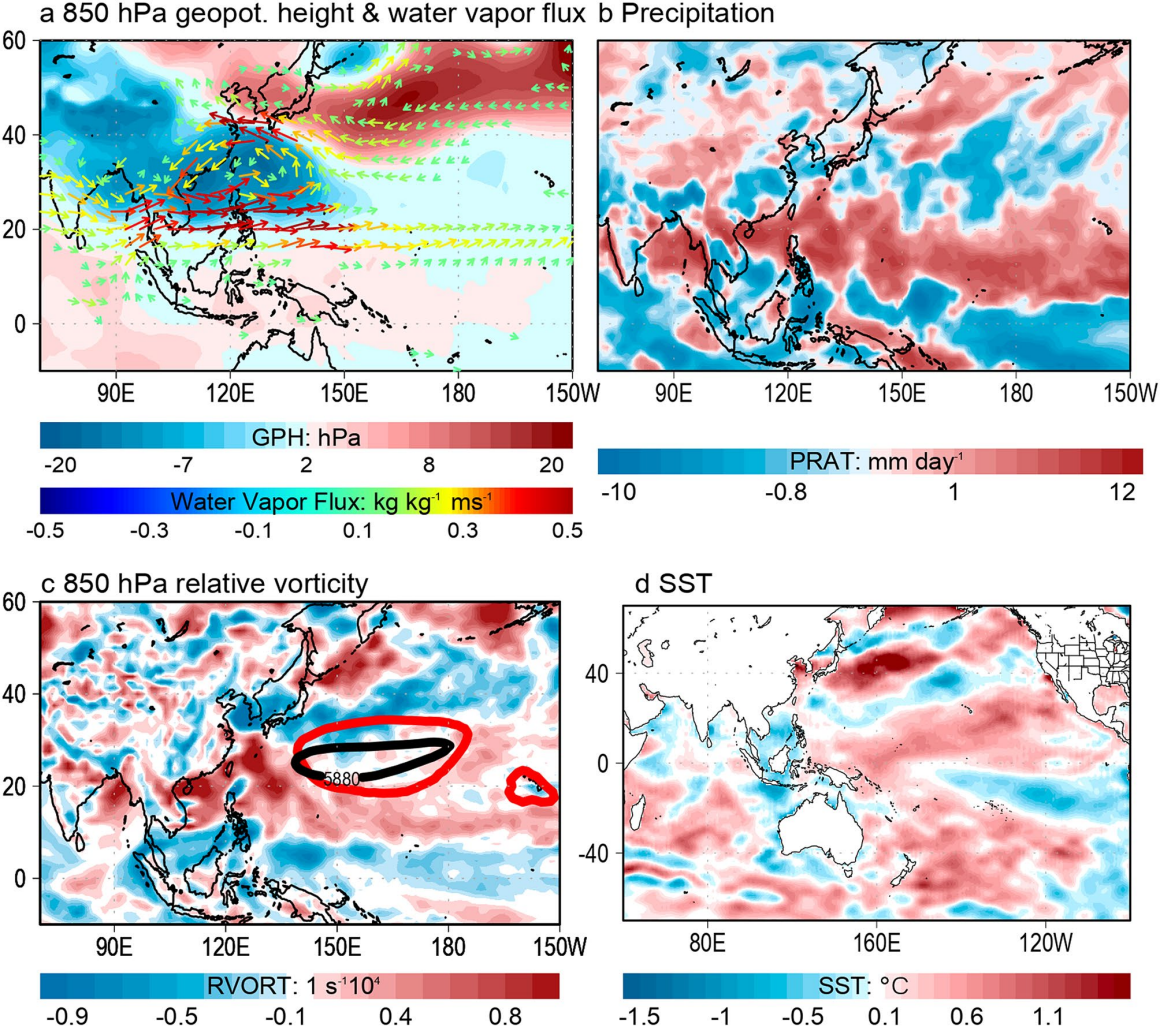
Anomalies in the large-scale environment during the 2018 boreal summer with respect to the 1984–2018 climatology. Anomalies in 850 hPa geopotential height (shaded) and water vapor flux (vector) (**a**), precipitation (**b**), 850 hPa relative vorticity (**c**), and sea surface temperature (**d**), respectively. In (**c**) the red and black contour lines represent the location of the Western North Pacific subtropical high during JJA 2018 and its climatology, respectively. The maps are plotted using GrADS v2.2.1 (http://opengrads.org/).

In JJA 2018, the WNP was highlighted by an anomalous low pressure region flanked by anomalous westerlies to its south and anomalous easterlies to its north, which converged in a zonally-elongated tropical convective activity, anomalous background cyclonic vorticity and wider WNP subtropical high (Fig. 2a–c). The warmer SST anomalies in the central Pacific siphoned the anomalous westerlies from the cool Indian Ocean SST anomalies prompting an extended anomalous cyclonic circulation to the central tropical Pacific. In addition, the anomalous subtropical easterlies originating from a weakened North Pacific High stalled and slowed down the total speed of subtropical westerlies, which further warmed the ocean surface off the coast of Japan (Fig. 2d). The warm SSTs in the subtropical and tropical WNP provided increased low-level water vapor flux resulting to a more humid environment for sustained convective activities^6^. When combined altogether, these anomalies characterize the highest TC frequency in JJA 2018.

In addition, we found that the WNP summer monsoon index (WNPMI)^6,7^ was also in its highest point in JJA 2018 (Fig. 1b) and it is surprising that there is inadequate literature about such unprecedented record. The implication of the said highest WNPMI intensity to the TC frequency in JJA 2018 is analyzed in our succeeding discussion.

### Influence of the large-scale environment to the WNP TC frequency variability

**Figure 3:**
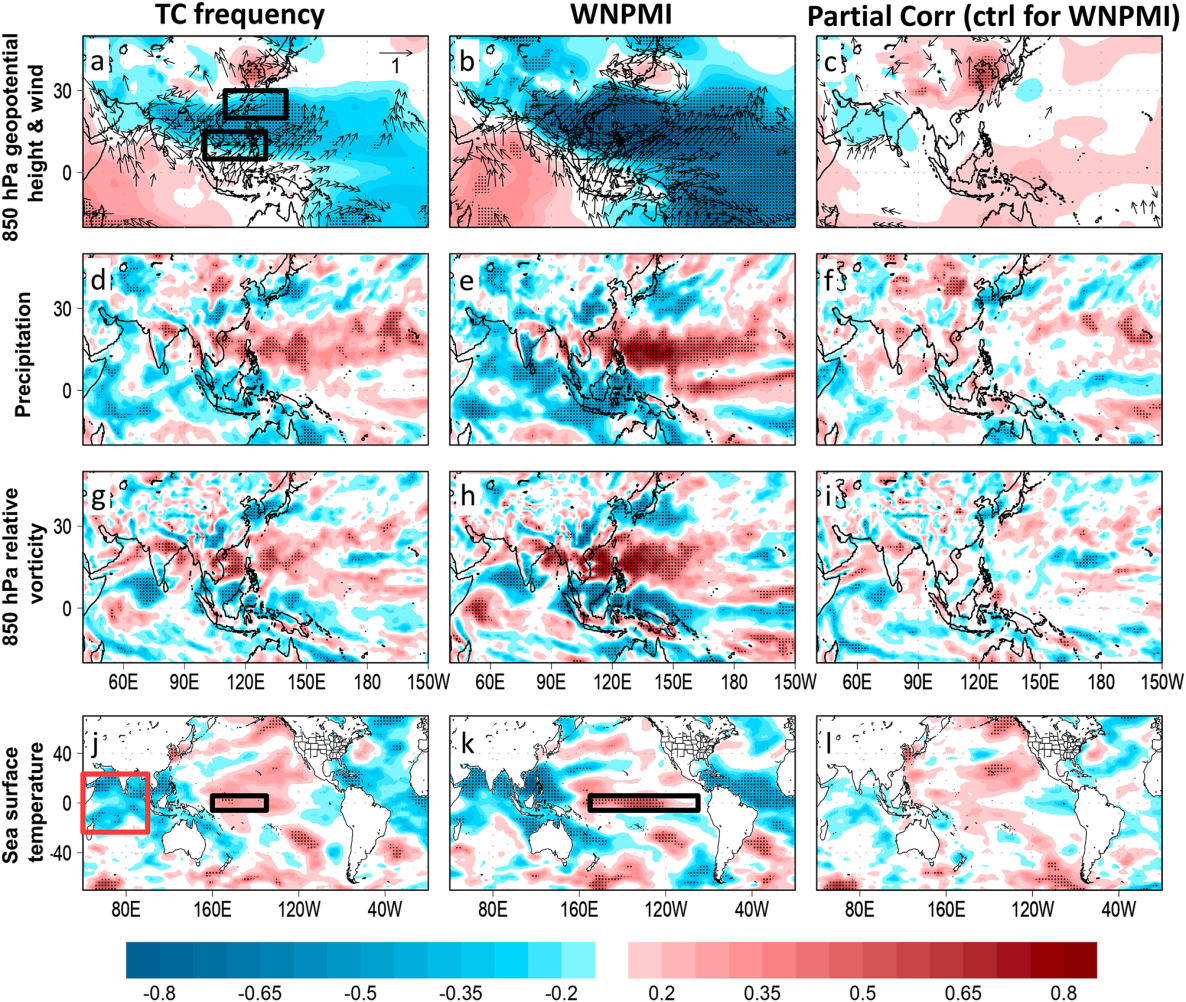
Spatial correlation maps of tropical cyclone (TC) frequency and the Western North Pacific (WNP) summer monsoon index against indicated large-scale environment variables. Left panel, spatial correlation maps of TC frequency and 850 hPa geopotential height (shaded) and wind (vector) (**a**), precipitation (**d**), 850 hPa relative vorticity (**g**), and sea surface temperature (**j**). Middle panel, same with the left panel but shows spatial correlations for the WNP summer monsoon index (WNPMI). Right panel, partial correlation maps of TC frequency and indicated variables when controlled for the WNPMI. In (**a**) the black boxes indicate the index locations of the WNP summer monsoon. In (**j**) the black (red) box represents the index location of the Central Pacific ENSO (Indian Ocean Basin Wide SST). In (**k**) the black box indicates the index location of the combined ENSO regions. In (**a**)–(**l**), the black dots represent significance of correlation at *p* < 0.05 confidence level. The maps are plotted using GrADS v2.2.1 (http://opengrads.org/).

To highlight the areas that modulate the WNP TC frequency variability in JJA, we plotted the spatial correlation of TC frequency and the large-scale environment (Fig. 3a,d,g,j). The major takeaways in the spatial correlation patterns include the strong westerlies over the Philippines and counter-flowing subtropical easterlies over Taiwan indicative of strong WNP summer monsoon, zonally elongated tropical convective activity, and the dipole SST pattern between the tropical Indian Ocean and the central Pacific Ocean. We note that the WNP TC frequency is significantly correlated with the following climate indices known to modulate WNP TC variability: WNPMI; r = 0.6, *p* = 0.000)^7,8^ (Fig. 1b), WNP subtropical high index (r = − 0.47, *p* = 0.004)^9^, and Indian Ocean Basin Wide SST (IOBW, r = − 0.48, *p* = 0.004)^10,11^ (Supplementary Fig. 2a,b). Meanwhile, the WNP TC frequency has only marginal correlation with the Pacific Meridional Mode^11^ (r = 0.34, *p* = 0.045) and the Niño 4 index (r = 0.31, *p* = 0.069)^12^ (Table 1; Supplementary Figs. 1b, 2c). We selected the Niño 4 index among the different ENSO indices (i.e. Niño 3.4, Niño 4, and Niño 3, Niño 1 + 2) because the Niño 4 index is closest Niño index to the WNP. The Indian Ocean Dipole index, which is another Indian Ocean SST-based index^10,11^, is practically not correlated with WNP TC frequency in JJA (r = 0.01) plus there is no distinguishable anomalous SST dipole pattern between the western and eastern tropical Indian Ocean in JJA 2018 (Fig. 2d). Between the WNPMI and the WNP subtropical high index, only the former registered its simultaneous highest record during the JJA 2018 (Fig. 1b; Supplementary Fig. S2a).

**Table 1:** Correlation and partial correlation coefficient of TC frequency and indicated climate index.

Index	WNPMI	IOBW	Niño 4	PMM	
**Zero-order bivariate correlation**					
TC frequency	0.60 **(0.000)**	− 0.48 **(0.004)**	0.31 (0.069**)**	0.34 **(0.045)**	
WNPMI		− 0.40 **(0.016)**	0.34 **(0.045)**	0.05 (0.772)	
IOBW			0.38 **(0.026)**	− 0.16 (0.372)	
Niño 4				0.33 (0.052)	

Control	Index	WNPMI	IOBW	Niño 4	PMM
**Partial correlation**					
WNPMI	TC frequency		− 0.33 (0.062)	0.14 (0.421)	0.39 **(0.023)**
IOBW		0.50 **(0.003)**		0.60 **(0.000)**	0.31 (0.077)
Niño 4		0.55 **(0.001)**	− 0.68 **(0.000)**		0.27 (0.128)
PMM		0.62 **(0.000)**	− 0.46 **(0.006)**	0.22 (0.204)	

Control	Index	TC frequency	IOBW	Niño 4	PMM
IOBW	WNPMI	0.50 **(0.003)**		0.58 **(0.000)**	− 0.01 (0.940)
Niño 4		0.55 **(0.001)**	− 0.61 **(0.000)**		− 0.07 (0.694)
PMM		0.62 **(0.000)**	− 0.46 **(0.006)**	0.22 (0.204)	

Values inside parentheses indicate the *p*-value. The *p*-value in bold is significant using two-tailed distribution test with 95% confidence.

It is reported that a positive IOBW contributes to the formation of an anticyclonic circulation in the WNP that suppresses convective activities particularly during July and August^13,14^. Furthermore, a positive IOBW is also found to force a surface divergence in the WNP emanating from an equatorial Kelvin wave that propagates into the WNP. A suppressed convective activity on top of an anomalous anticyclonic circulation and low-level divergence in the WNP are unfavorable environment for increased TC development in the WNP. Meanwhile, it is widely thought that warm tropical Pacific SSTs (e.g. Niño 4) induce increased surface fluxes, anomalous low-level westerlies and stronger vertical upward motion, which enhance pre-existing convective activities necessary for TC development^15–17^. Furthermore, a warm PMM contributes to an increased WNP TC frequency by prompting a weaker vertical wind shear and increased relative vorticity, primarily in the Southeastern portion of the WNP, which generally result to more TC development in the WNP^18,19^. The influence of WNPMI to TC frequency is discussed in the following section.

We performed partial correlation analysis to test the influence of WNPMI, IOBW, Niño 4, and PMM to the WNP TC frequency (Table 1). When the WNPMI is controlled, the correlation between the TC frequency and IOBW (r = − 0.33, *p* = 0.062) and PMM (r = 0.39, *p* = 0.023) becomes marginally significant, and insignificant with the Niño 4 index (r = 0.14, *p* = 0.421), respectively. When the IOBW, Niño 4, and PMM are individually controlled, the partial correlations between the WNPMI and TC frequency remains significant at r = 0.50 (*p* = 0.003), r = 0.55 (*p* = 0.001) and r = 0.62 (*p* = 0.000), respectively, which suggest that the WNPMI controls the variability of TC frequency more than the IOBW, Niño 4, and PMM.

Moreover, when the IOBW is controlled, the partial correlation between TC frequency and Niño 4 becomes significant at r = 0.60 (*p* = 0.000) and marginally significant with the PMM (r = 0.31, *p* = 0.077). When the PMM is controlled, the partial correlation between TC frequency and IOBW remains significant (r = − 0.46, p = 0.006) and insignificant with Niño 4 index (r = 0.22, *p* = 0.204). Lastly, when the Niño 4 index is controlled the partial correlation between TC frequency and IOBW remains significant (r = − 0.68, p = 0.000) and insignificant with the PMM (r = 0.27), which means that the IOBW has higher influence to TC frequency than the Niño 4 index and PMM, respectively. The higher partial correlation of both Niño 4 and IOBW is anchored on the fact that they have contrasting correlation signal with TC frequency hence when the influence of the other is controlled their respective zero-order correlation with TC frequency becomes higher.

**Figure 4:**
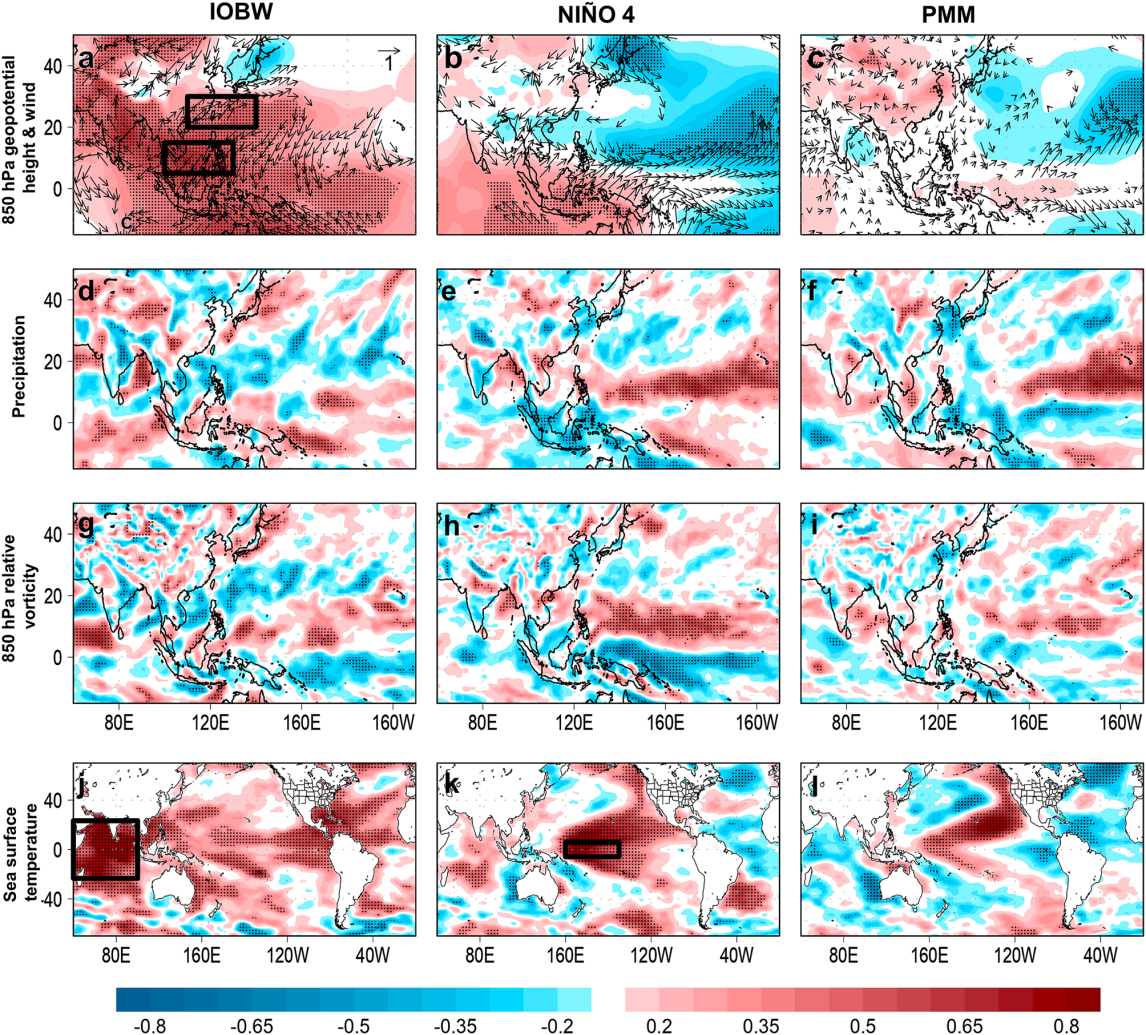
Spatial correlation maps of indicated climate index and large-scale environment. Left panel, spatial correlation maps of Indian Ocean Basin Wide (IOBW) index and 850 hPa geopotential height (shaded) and wind (vector) (**a**), precipitation (**c**), 850 hPa relative vorticity (**e**), and sea surface temperature (**d**). Middle to right panel, same with the left panel but shows Niño 4 and the Pacific Meridional Mode (PMM) indices, respectively. In (**a**) the black boxes indicate the index locations of the WNP summer monsoon index. In (**j**,**k**) the black box represents the index location of the IOBW and Niño 4, respectively. In (**a**)–(**l**) the black dots represent significance of correlation at *p* < 0.05 confidence level. The maps are plotted using GrADS v2.2.1 (http://opengrads.org/).

The spatial correlation patterns of IOBW show significant positive correlation with an anomalous anticyclonic circulation over the Philippines, weak monsoon flow and convective activities, deeper region of cyclonic vorticity, and warm tropical Indian and central Pacific SSTs (Fig. 4a,d,g,j); the latter explains why the IOBW is significantly correlated with the Niño 4 index (r = 0.38, *p* = 0.026) and not with PMM (r = − 0.16) (Table 1).

Most studies on similar subject reported that the central Pacific SST warming and positive Pacific Meridional Mode (PMM) has influenced the highest TC frequency in JJA 2018^1–4^. We show that a warm Niño 4 is correlated with anomalous convective activities and anomalous cyclonic vorticity that are closer to the monsoon region (Fig. 4b,e,h,k) while a warm PMM phase is more correlated with anomalous convective activities and anomalous cyclonic vorticity located at around 160°E (Fig. 4c,f,i,l), which is the reason why the WNPMI is significantly correlated with the Niño 4 (r = 0.38, *p* = 0.045) and is practically not correlated with the PMM (r = − 0.05). It is previously demonstrated that the PMM is more correlated with TC genesis at around 160°E and is not correlated with TC genesis westward of 160°E^12^. In JJA 2018, the mean TC genesis point is located around 140°E (Fig. 1a) and the most intense anomalous cyclonic vorticity is located over the Philippines (Fig. 2a–c), which is more proximal to the most intense convection center associated with the Niño 4 and is beyond the convection center associated with the PMM. Therefore, in the context of the highest TC frequency in JJA 2018, the Niño 4 may have more influence than the PMM. Such influence is further confirmed when the PMM-related SST warming is separated from the central Pacific SST-related warming during the JJA 2018 in the WNP^4^. It is shown that the PMM-related SST warming in JJA 2018 resulted to weaker tropical convection while the central Pacific SST-related warming induced strong tropical convection and anomalous cyclonic circulation over most parts of the WNP. With this, we support the report of^4^ by concluding that the central Pacific SST exerted more influence than the PMM in characterizing the increased TC frequency in JJA 2018.

Both IOBW and the Niño 4 did not reach their extreme values nor are they unusually abnormal in JJA 2018 (Supplementary Fig. S2b,c). However, we suspect that the combined influence of these two climate indices resulted to the highest TC frequency record (including the highest WNPMI) because there are only three simultaneous years when the IOBW was cooler than the 1st interquartile range value of its climatology while the Niño 4 index was simultaneously warmer than the 3rd interquartile range value of its climatology. These years include 1994 (16 TCs), 2004 (15 TCs), and 2018 (17 TCs) corresponding to the three years with the highest WNP TC frequency in JJA (Supplementary Fig. S2b,c). We also note that the WNPMI values in these years were also simultaneously above the 3rd interquartile range value percentile of its climatology.

### Simultaneous highest WNP summer monsoon intensity

It is surprising that there is inadequate discussion on the simultaneous highest WNPMI intensity in JJA 2018 among the existing reports on the highest records in TC frequency (Fig. 1b). We previously stressed that the WNPMI and WNP TC frequency both observed their highest and lowest records in JJA 2018 and 1998, respectively. The WNP summer monsoon is characterized by a low pressure region centered over the Philippines, anomalous westerlies and cross-equatorial southwesterlies, prominent tropical convective heat source and anomalous cyclonic vorticity to the east of the Philippines, cool IOBW SST, and warm central Pacific SST (Fig. 3b,e,h,k). The WNPMI is a measure of the intensity of the WNP summer monsoon defined by taking the difference of the mean 850 hPa zonal wind speed in the two regions of the WNP (Fig. 3a)^6,7^. The prominent similarities in the spatial correlation patterns of the WNP TC frequency and the WNPMI with the corresponding large-scale environment suggest that when the WNP summer monsoon is more intense, there is expectedly higher WNP TC frequency during JJA. To iterate from our previous discussion, we found that the partial correlation of the WNPMI and TC frequency remains significant even without the influence of the other indicate indices.

**Figure 5:**
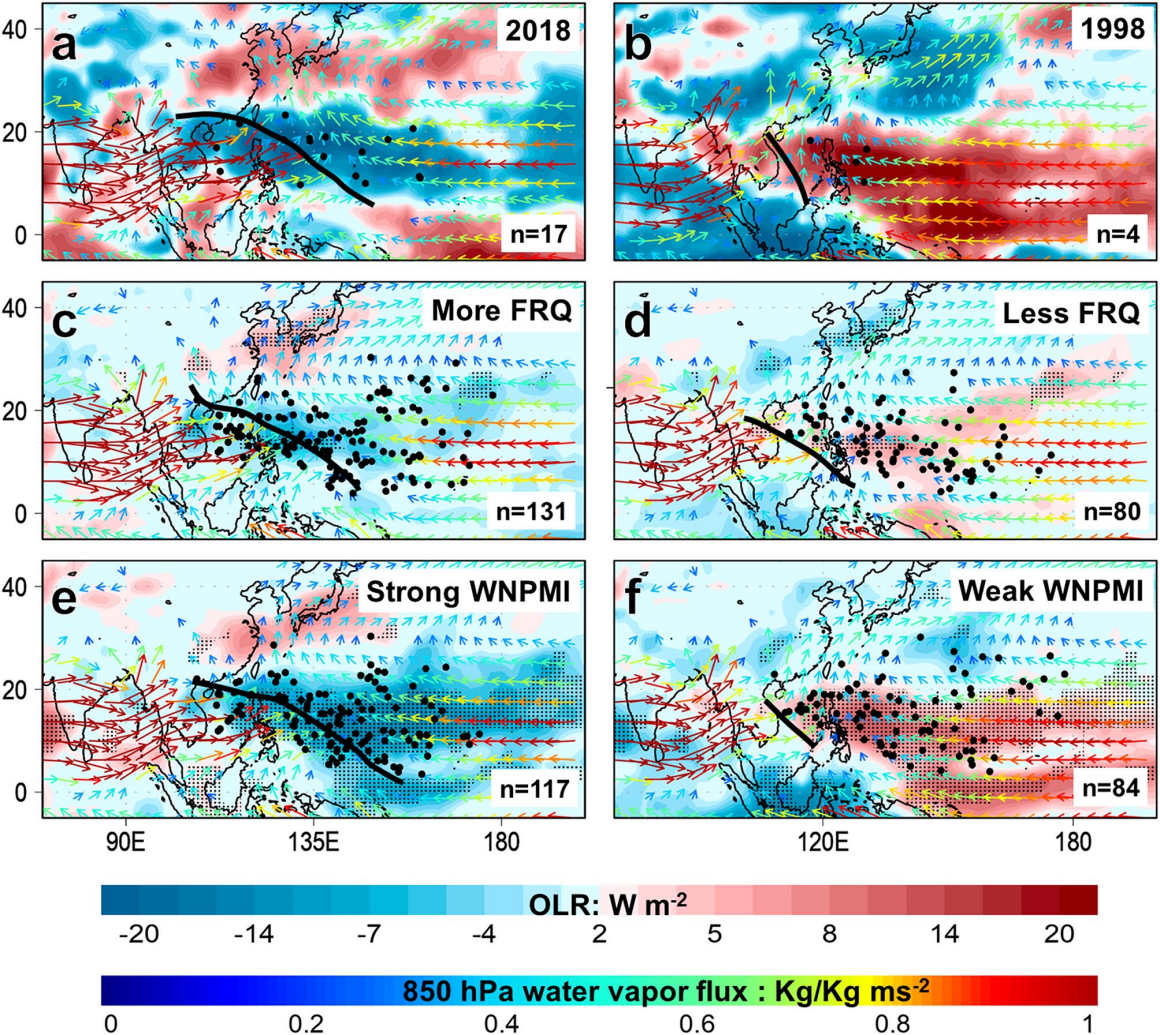
Composite difference maps associated with the tropical cyclone (TC) frequency and the Western North Pacific (WNP) summer monsoon index (WNPMI). Left panel, composite difference maps of outgoing longwave radiation (shaded) and 850 hPa water vapor flux (vector) in JJA 2018 (**a**), years with more TC frequency (**c**), and strong WNPMI (**e**), respectively. Right panel, same with the left panel but shows JJA 1998 (**b**), less TC frequency (**d**), weak WNPMI (**f**), respectively. In (**a**)–(**f**), the inset n-value indicates the total TC frequency of their respective composite years. The black circles and black lines represent the location of TC genesis points and the monsoon trough during the indicated composite years, respectively. In (**c**)–(**f**) the black dots represent significance of difference at *p* < 0.05 confidence level. The maps are plotted using GrADS v2.2.1 (http://opengrads.org/).

We plotted the composite difference in mean seasonal outgoing longwave radiation (OLR) and 850 hPa wind anomalies in JJA 2018 and 1998, years with strong and weak WNPMI, and years with more and less TC frequency, respectively (Fig. 5a–f). Strong convective activities marked by negative OLR anomalies and extended monsoon trough dominate the tropical WNP in JJA 2018, years with more TC frequency, and years of strong WNPMI while weak convective activities and shorter monsoon trough underscored by positive OLR anomalies are prominent in JJA 1998, years with less TC frequency, and years of weak WNPMI. In JJA 2018, years with high TC frequency, and strong WNPMI, the increased anomalous westerlies are drawn towards the convective heat source in the central Pacific and converge with the anomalous easterlies along the eastward-extended monsoon trough where more TCs developed. An extended monsoon trough provides moist environment that reduces entrainment of dry air, and increased contiguity of background cyclonic vorticity that bands pre-existing tropical convective disturbances together, which is why about 60–90% of TCs in the WNP in JJA develop in vicinity of the monsoon trough^20^. These composite maps support that the strong WNPMI in JJA 2018 has significantly and simultaneously influenced the highest TC frequency record in JJA 2018. To further test such inference, we plotted the spatial partial correlation of TC frequency while controlling for the influence of the WNPMI. After performing partial correlation, the areas that are originally and significantly correlated with TC frequency became practically absent; this confirms that the WNP summer monsoon modulates WNP TC frequency in JJA (Fig. 3c,f,i,l).

One remaining open question is, between the IOBW and Niño 4, which has more influence to the WNPMI? The WNPMI is more significantly correlated with the IOBW (r = − 0.4, *p* = 0.016) than the Niño 4 (r = 0.34, *p* = 0.045) (Table 1). The partial correlation between the WNPMI and IOBW while controlling for the Niño 4 is r = 0.61 (*p* = 0.000), and the partial correlation between the WNPMI and Niño 4 while controlling for IOBW is r = 0.58 (*p* = 0.001). The test of power of correlation between IOBW and Niño 4 suggests that there is 94.6% chance that the correlation coefficients are similar with each other and the risk of committing a Type II error while such similarity is false is only 5.4%. Therefore, both IOBW and Niño 4 have similarly influenced the WNPMI. We recognize that the WNP summer monsoon is also modulated by a variety of other factors such as the Intertropical Convergence Zone (ITCZ)^21^, WNP subtropical high, interhemispheric and regional thermal contrasts (which also drives the ITCZ), but such matter is another subject for future investigation.

## Discussion and conclusion

At the intraseasonal scale, the time-longitude diagram of 5-day running mean filtered daily 850 hPa zonal wind and OLR anomalies averaged from 10°S-10°N in JJA 2018 indicate enhanced anomalous westerlies and anomalous deep tropical convection throughout the WNP (Supplementary Fig. 3a,b). The Madden–Julian Oscillation (MJO) is an intraseasonal tropical mode characterized by an eastward-propagating system of tropical convective activities that travels through its different phases; it is nearest to the maritime continent and WNP during its Phases 5–6. Correspondingly, the MJO was active when it was located in its Phases 5–6, which means that there exist stronger anomalous westerlies and negative outgoing longwave radiation anomalies in the WNP at the same phase^22,23^ that are favorable precursors for TC development. In addition, the Boreal Summer Intraseasonal Oscillation (BSISO), which is another intraseasonal mode of tropical convective activity in the Asian monsoon region, was also active when it was located in its Phases 2 and 8 (nearest to the Philippine Sea and WNP)^24^. These active tropical convective activities imply that the intraseasonal modes may have concurrently influenced the extreme TC record in JJA 2018 frequency. In addition, most TC genesis points are embedded in the daily OLR and 850 hPa zonal winds in JJA 2018 (Supplementary Fig. 3a,b).

In longer timescales, the WNP TC frequency in JJA is shown to have a high frequency variability of 3–4 years and low frequency periodicity every 10–12 years; such low frequency variability implies that multiyear modes also influence TC frequency in the WNP (Supplementary Fig. S4). The reported correlation stability between the WNPMI and TC frequency beginning in 1998 due to the shift of Pacific Decadal Oscillation to its negative phase^25^ further supports the idea that the long-term modes of variability also influences WNP TC frequency.

**Figure 6:**
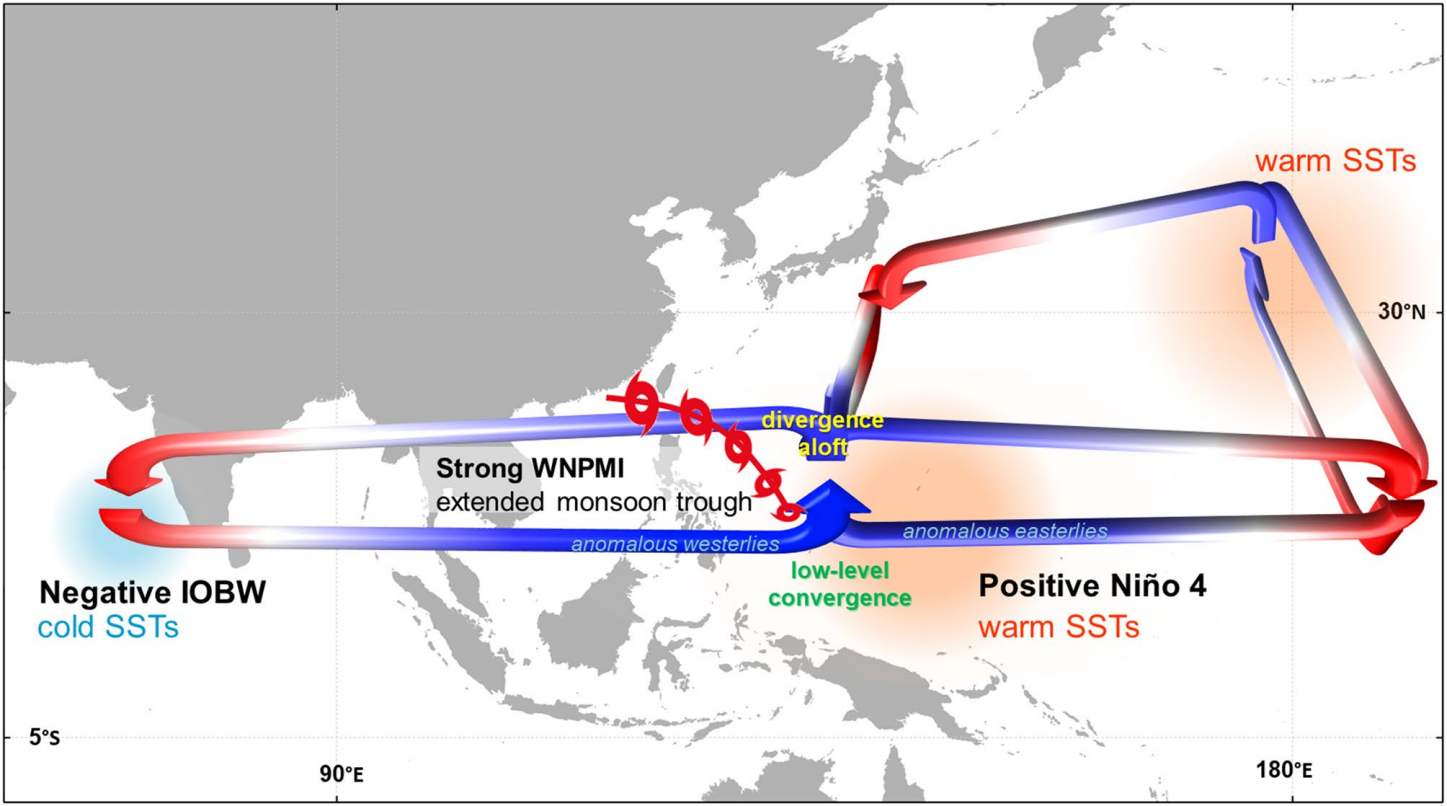
Schematic of the highest tropical cyclone (TC) frequency during the 2018 boreal summer. The arrows indicate the direction of the circulation while the blue (red) color represents low (high) pressure regions. The anomalous anticyclonic circulation and cold sea surface temperature (SST) anomalies in the tropical Indian Ocean prompt anomalous westerlies to flow and converge with easterlies towards the warm central Pacific Ocean, which eventually rises as an anomalous convective activity and meridionally descends as dry air in the WNP subtropical high and in the eastern Pacific Ocean. The zonally descending air in the eastern Pacific jumpstarts its region’s meridional circulation, which consequentially rises to a weakened North Pacific High, generates anomalous subtropical easterlies, stalls the subtropical jetstream, and prompts warm SST anomalies off the coast of Japan. The warm SSTs in the tropical and subtropical WNP lead to an increased background moist environment and cyclonic vorticity, which ultimately becomes favorable environment for increased TC development in the WNP. The schematic is drawn using Microsoft PowerPoint (https://www.microsoft.com/).

We deviate from the previous reports by demonstrating that the recorded highest TC frequency in JJA 2018 in the WNP is primarily attributed with the simultaneous recorded highest intensity of the WNPMI. Observational evidence were showed that the combined influences of the cool IOBW and the warm Niño 4 have similarly modulated the simultaneous highest recorded TC frequency and the highest recorded intensity of the WNPMI, which are held true for the three years (i.e. 1994, 2004, and 2018) with highest WNP TC frequency and most intense WNPMI. The large-scale circulation patterns in JJA 2018 that prompted the recorded highest TC frequency during can be summarized as follows: a cool tropical Indian Ocean SST (negative IOBW) is associated with an anomalous anticyclonic circulation in the Indian Ocean, which generates low-level anomalous westerlies drawn towards the anomalous convective heat source in the central Pacific associated with warm central Pacific SST anomalies (positive Niño 4) (Fig. 6). The strengthened anomalous westerlies converge with the trade winds and prompt an extended monsoon trough (positive WNPMI), which increases low-level water vapor flux and contiguity of cyclonic vorticity, and enhances deep tropical convective activities. When combined altogether, these anomalies in the large-scale environment become conducive for increased TC development in the WNP in JJA 2018. Furthermore, the risen moist air in the central Pacific zonally (meridionally) descends as dry air in the eastern Pacific (subtropical WNP). In the eastern Pacific, the sunken dry air mass meridionally rises as moist air thus weakening the North Pacific High where it slows down the total speed of the subtropical westerlies leading to warm SSTs off the coast of Japan; this generates surface necessary to sustain TC passages in this region. The overturning subtropical easterlies from the anomalous WNP cyclonic circulation also contribute to the slowdown of the subtropical westerlies. Completing the cycle, the anomalous easterlies from the eastern Pacific converge with the anomalous westerlies along the extended monsoon trough in the WNP.

We also support that the positive PMM generally poses secondary roles to TC development in the WNP, especially in the context of the highest TC frequency in JJA 2018, but not with the simultaneous highest WNPMI intensity. We also note that the PMM is more correlated with the TC frequency in the Eastern North Pacific (r = 0.42, *p* = 0.012) and the combined TC frequency in the WNP and the Eastern North Pacific (r = 0.49, *p* = 0.003) in JJA than the WNP TC frequency alone (Supplementary Fig. S1a,b), which further supports that the PMM is more important in modulating TC frequency in the Eastern North Pacific than in most parts of the WNP^3,4^.

Our study contributes additional insights to existing knowledge pool to characterize the highest tropical cyclone frequency in the Western North Pacific. Ultimately, it is expected that such contribution supports process understanding of extreme TC events.

## Methods

### Tropical cyclone best track and reanalysis dataset

The TC best track data are obtained from the International Best Track Archive for Climate Stewardship version 4 from 1984–2018^26^ in the WNP. Our period of analysis covers the period 1984–2018 parallel to the reported reliable years during the satellite period of TC observations in the WNP^27^. Only named tropical cyclones with maximum sustained winds that are greater than or equal to 35 knots (~ 17 ms^−1^) were considered in our analysis. Similar rules were applied for the counting of TC frequency in the Eastern North Pacific.

We used the Japanese Reanalysis 55-year Project from 1984–2018 in our observational analysis^28^. In the composite analysis, a year with more (less) TC frequency is defined as a year that is greater than or equal (less than or equal) to the 75th (25th) percentile range of the base period (1984–2018). Same definition of percentile rank is used for the strong and weak WNPMI. The years with more TCs include 1989, 1992, 1994, 1997, 2001, 2004, 2013, 2017, and 2018 while the years with less TCs are 1986, 1987, 1995, 1998, 1999, 2003, 2007, 2008, 2010, 2014, and 2015. Meanwhile, the years with strong WNPMI are 1985, 1986, 1990, 1994, 2001, 2002, 2004, 2012, and 2018 while the years with weak WNPMI are 1988, 1993, 1995, 1996, 1998, 2007, 2008, 2010, and 2017.

### Climate indices and other metrics

The climate indices used in our analysis are defined as follows:

- Western North Pacific Monsoon Index—difference of zonal winds at 850-hPa between a southern region (5°–15°N, 100°–130°E) and a northern region (20°–30°N, 110°–140°E) ^6,7^. The location of the monsoon trough is identified using the overturning 0 contour line of 850 hPa zonal wind in the WNP.
- Indian Ocean Basin Wide SST—areal-averaged JJA SST at 20°S–20°N, 40–100°E^9,10^
- Niño 4 index—areal-averaged JJA SST anomalies at 5°S–5°N, 160–210°E^11^. We used the Niño 4 index as the ENSO flavor of our analysis because it is the most proximal classic Niño region to the WNP.
- Pacific Meridional Mode index—maximum covariance mode of meridional SST in the Pacific ^11^
- Western North Pacific subtropical high index—areal-averaged 850 hPa geopotential height at 15–25°N, 115–150°E ^8^. The location of the WNP subtropical high index is identified using the 5880 gpm contour line.

### Statistical tests

The Pearson’s correlation is the selected measure of bivariate correlation and partial correlation in our study where their significance is tested using t-test with two-tailed distribution. The significance of difference is testing using student’s t-test. We applied 1,000 simulations in the student’s t-test using Monte Carlo Test to minimize the influence of uncertainty in the significance testing. The power for comparing two correlation coefficients was tested using Fisher-Z transformation.

## Supplementary Information

Supplementary Figures.

## Data Availability

The reanalysis data products and climate indices used in the analysis are available for download from their respective websites.
