# Supplementary material for: Characterizing the highest tropical cyclone frequency in the Western North Pacific since 1984
Source: Sci Rep. 2021 Jul 12;11:14350. doi: 10.1038/s41598-021-93824-2 (PMC8275583; doi:10.1038/s41598-021-93824-2)
Supplement: Supplementary file 1 — Supplementary Figures. [file 41598_2021_93824_MOESM1_ESM.docx]

**Characterizing the highest tropical cyclone frequency in the Western North Pacific since 1984**

**Joseph Basconcillo^1, 2, +^, Eun-Jeong Cha^3^, Il-Ju Moon^1,*, +^**

^1^Typhoon Research Center, Jeju National University, Jeju, South Korea

^2^Philippine Atmospheric, Geophysical, and Astronomical Services Administration, Department of Science and Technology, Quezon City, Philippines

^3^National Institute of Meteorological Sciences, Jeju, South Korea

^*^ [ijmoon@jejunu.ac.kr](mailto:ijmoon@jejunu.ac.kr)

**^+^** these authors contributed equally

**Supplementary Information**

This Supplementary Information includes:

Supplementary Figs. S1-S4

**
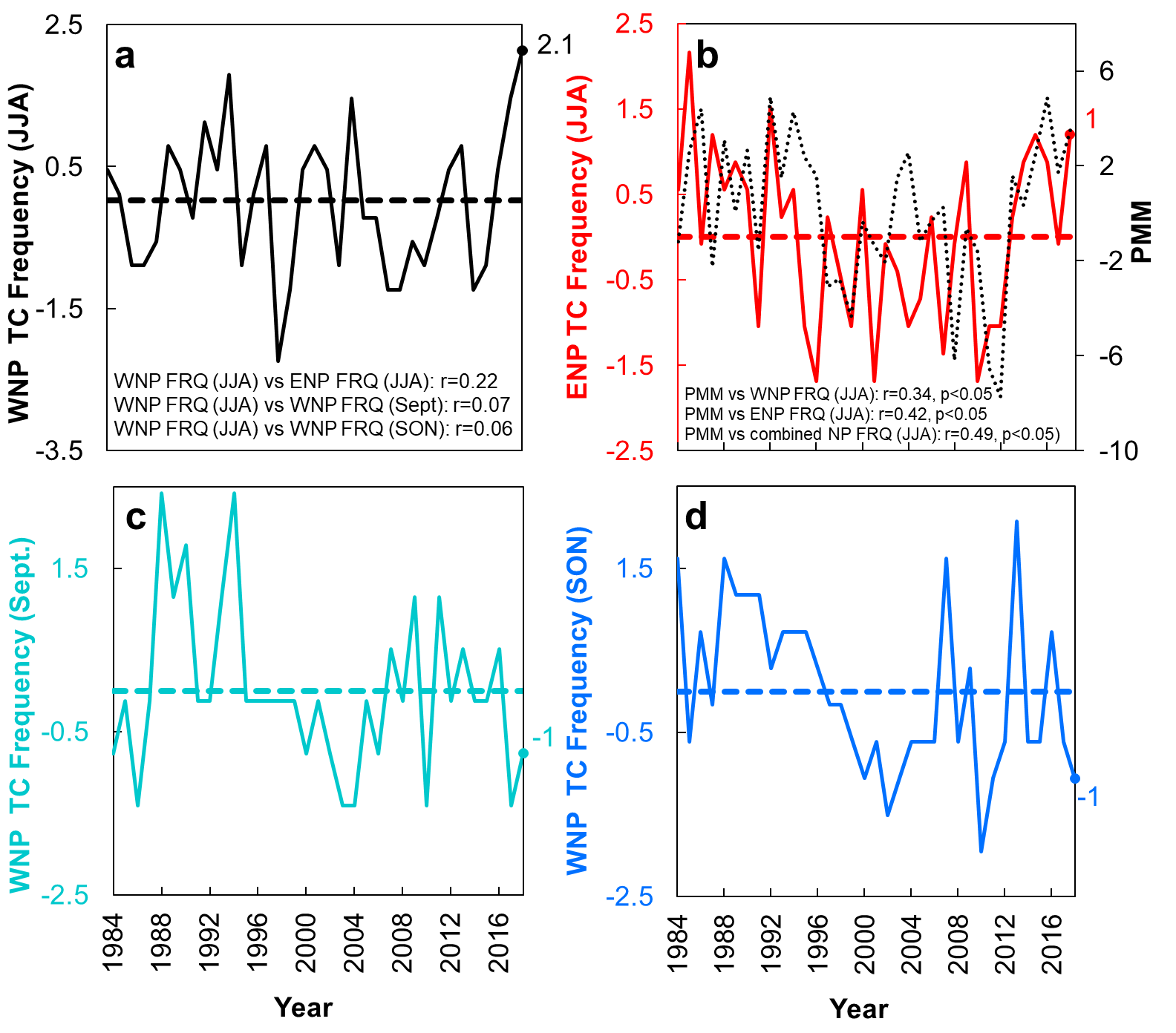
Supplementary Figure S1. Choice of scaling of tropical cyclone (TC) frequency. a-b,** Timeseries of standardized TC frequency anomaly in June-August (JJA) in the Western North Pacific (WNP) and in the Eastern North Pacific (ENP, red), respectively. **c-d**, Timeseries of standardized TC frequency anomaly in the WNP in September and in September-October (SON), respectively. In **a**, the inset statistics indicate the correlation of the WNP TC frequency in JJA and indicated TC frequency timeseries. In **b**, the black dotted line represents the timeseries of the Pacific Meridional Mode (PMM) while the inset statistics show the correlation of PMM and the indicated TC frequency timeseries. In **a-d**, the dashed lines indicate the climatology of each timeseries while the black dots and labeled numbers indicate their respective TC frequency in 2018.

**
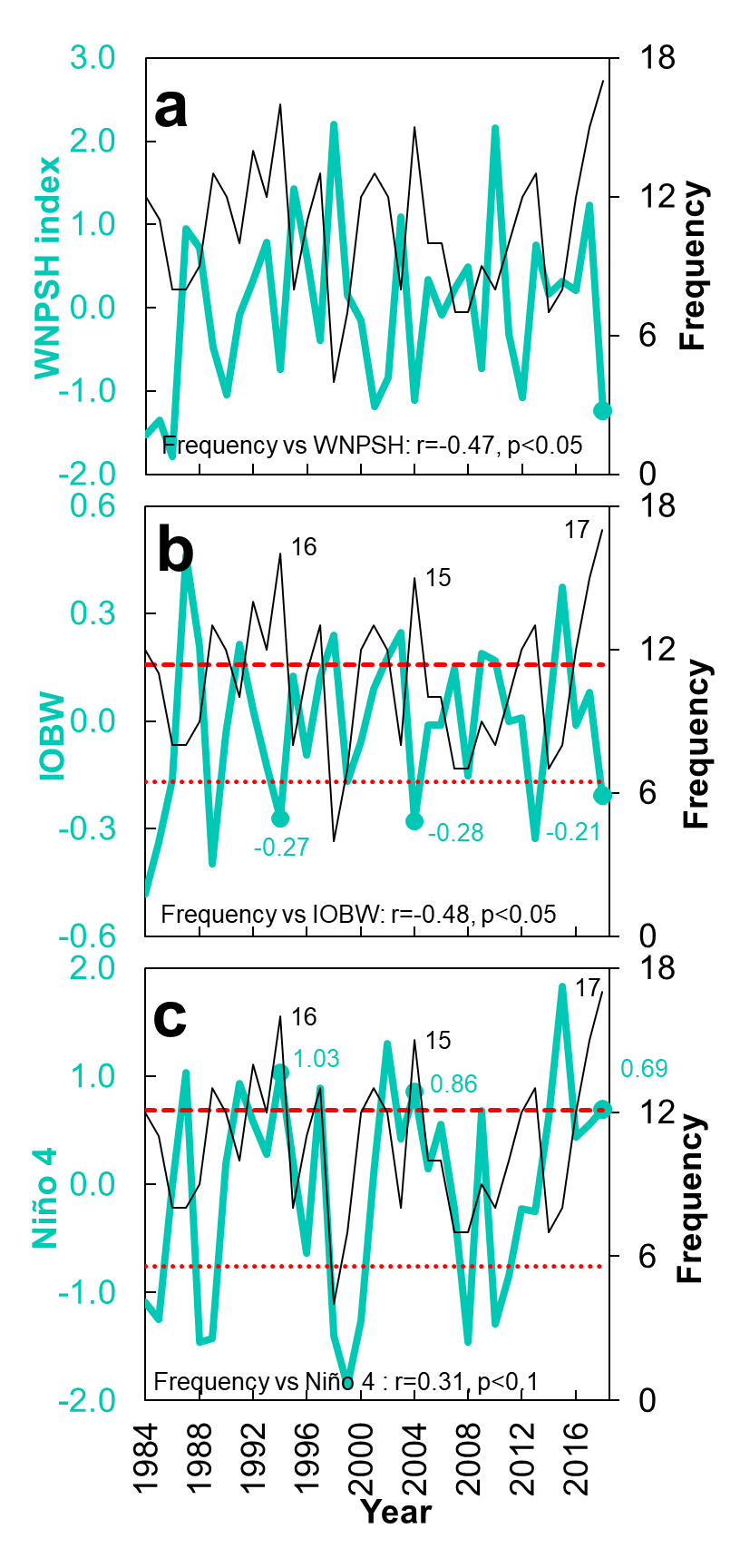
Supplementary Figure S2. Timeseries of the indicated climate index and tropical cyclone (TC) frequency during the boreal summer in the Western North Pacific (WNP). a,** Timeseries of TC frequency during the boreal summer (black) and the WNP subtropical high index (WNPSH, cyan) SST. **b-c**, same with **a** but shows the Indian Ocean Basin Wide (IOBW) and the Niño 4 indices, respectively. In **a-c**, the inset statistics indicate the correlation of the WNP TC frequency in JJA and the indicated index. In **b-c**, the red dashed (dotted) line represents the 75^th^ (25^th^) percentile of the indicated climate index. The labeled dots represent the indicated index value in 1994, 2004, and 2018, respectively.

**
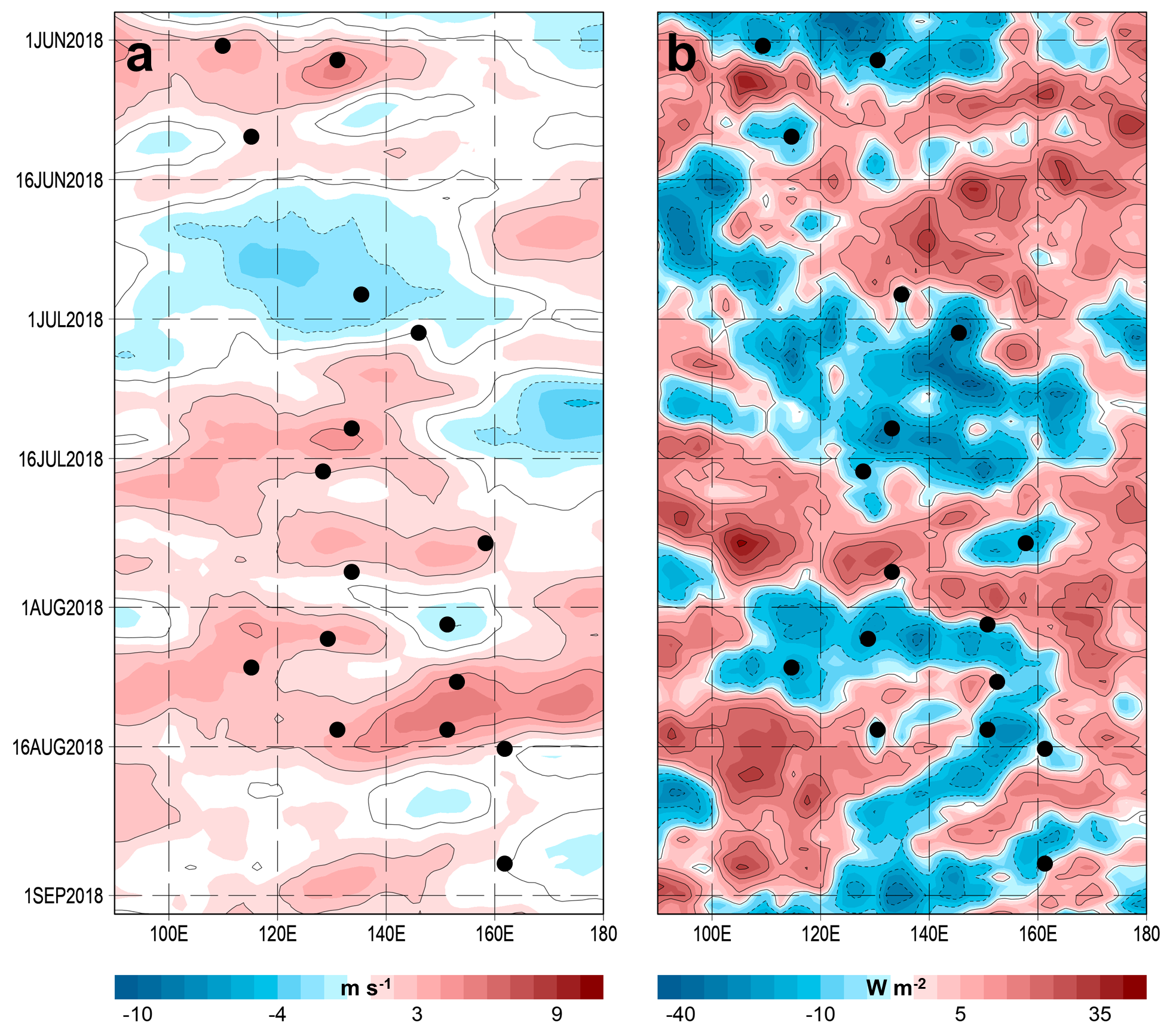
**

**Supplementary Figure S3. Influence of intraseasonal oscillations to WNP tropical cyclone (TC) frequency during the boreal summer**. **a-b**, Time-longitude diagram of 5-day running mean filtered daily zonal wind and outgoing longwave radiation (OLR) anomalies averaged over -10°S-10°N, respectively. The black dots represent the TC genesis points. Left to right-oriented diagonal positive (negative) zonal wind (OLR) anomalies indicate anomalous westerlies (convective activities).

**
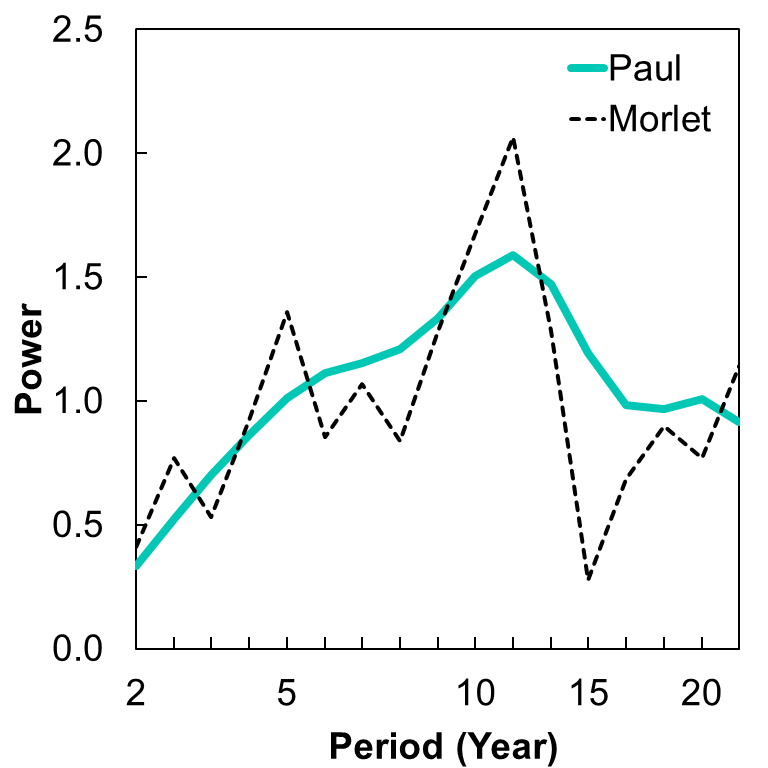
Supplementary Figure S4. Wavelet transform of the timeseries of tropical cyclone (TC) frequency in the Western North Pacific (WNP) during the boreal summer**. The black (cyan) line indicates the periodicity of the WNP TC frequency during the boreal summer using Morlet (Paul) wavelet transform. The WNP TC frequency during JJA has high frequency variability every 3-4 years and low frequency periodicity around 10-12 years.
